# Primary diffuse large B-cell lymphoma of the prostate: a case report and review of the literature

**DOI:** 10.1186/s13256-021-03143-3

**Published:** 2021-11-03

**Authors:** Mengwei Ren, Yanli Liu

**Affiliations:** 1 Department of Hematology and Oncology, the Second Affiliated Hospital of Inner Mongolia University for the Nationalities, Inner Mongolia General Forestry Hospital, No. 81, Lincheng Road, Yakeshi, Hulunbuir, 022150 Inner Mongolia Autonomous Region China; 2 Department of Medical Network Service, the Second Affiliated Hospital of Inner Mongolia University for the Nationalities, Inner Mongolia General Forestry Hospital, Yakeshi, Inner Mongolia Autonomous Region China

**Keywords:** Prostate, Diffuse large B-cell lymphoma, Lower urinary tract symptoms, Case report

## Abstract

**Background:**

Primary lymphoma of the prostate is an exceedingly rare disease, with diffuse large B-cell lymphoma being the most common known subtype in a small number of reported cases. Due to its low prevalence, there has been a chronic lack of targeted diagnostic guidelines and treatment procedures.

**Case presentation:**

In this article, we report a case of primary diffuse large B-cell lymphoma of the prostate in a 70-year-old Asian man who presented with symptoms of urinary tract obstruction. Histological and immunocytochemical studies of transurethral biopsy of the prostate showed diffuse large B-cell lymphoma. The patient was managed by a combination of eight courses of chemotherapy with a regimen including rituximab, cyclophosphamide, doxorubicin, vincristine, and prednisone and radiotherapy. Post-chemotherapy computed tomography scans showed complete remission. He remained disease free, until now, 15 months after the end of therapy. We also reviewed and analyzed relevant literature to illustrate the diagnosis, treatment, and prognosis of this disease.

**Conclusion:**

Diffuse large B-cell non-Hodgkin’s lymphoma originating in the prostate is a rare and highly aggressive disease that lacks specificity in its clinical presentation and is easily misdiagnosed. This disease should be considered clinically in patients with significant prostate enlargement and insignificant prostate-specific antigen elevation. The diagnosis can be clarified with a prostate puncture biopsy. Chemotherapy is the main treatment for patients and may be supplemented with surgical treatment and radiotherapy.

## Background

Primary lymphoma of the prostate is a relatively rare malignant tumor affecting the prostate gland, accounting for 0.09% of prostate neoplasms and only 0.1% of newly diagnosed lymphomas [1]. The majority of previously reported cases are diffuse large B-cell non-Hodgkin subtypes. Most patients are prone to suffer from lower urinary tract symptoms, and early imaging findings are similar to those of prostate cancer, which may lead to preoperative misdiagnosis. In this article, we report a case of primary diffuse large B-cell lymphoma (DLBCL) of the prostate managed with eight cycles of rituximab-based chemotherapy and local radiotherapy, with a review of the related literature to explore the diagnosis, treatment, and prognosis of this rare disease.

## Case report

**Fig. 1:**
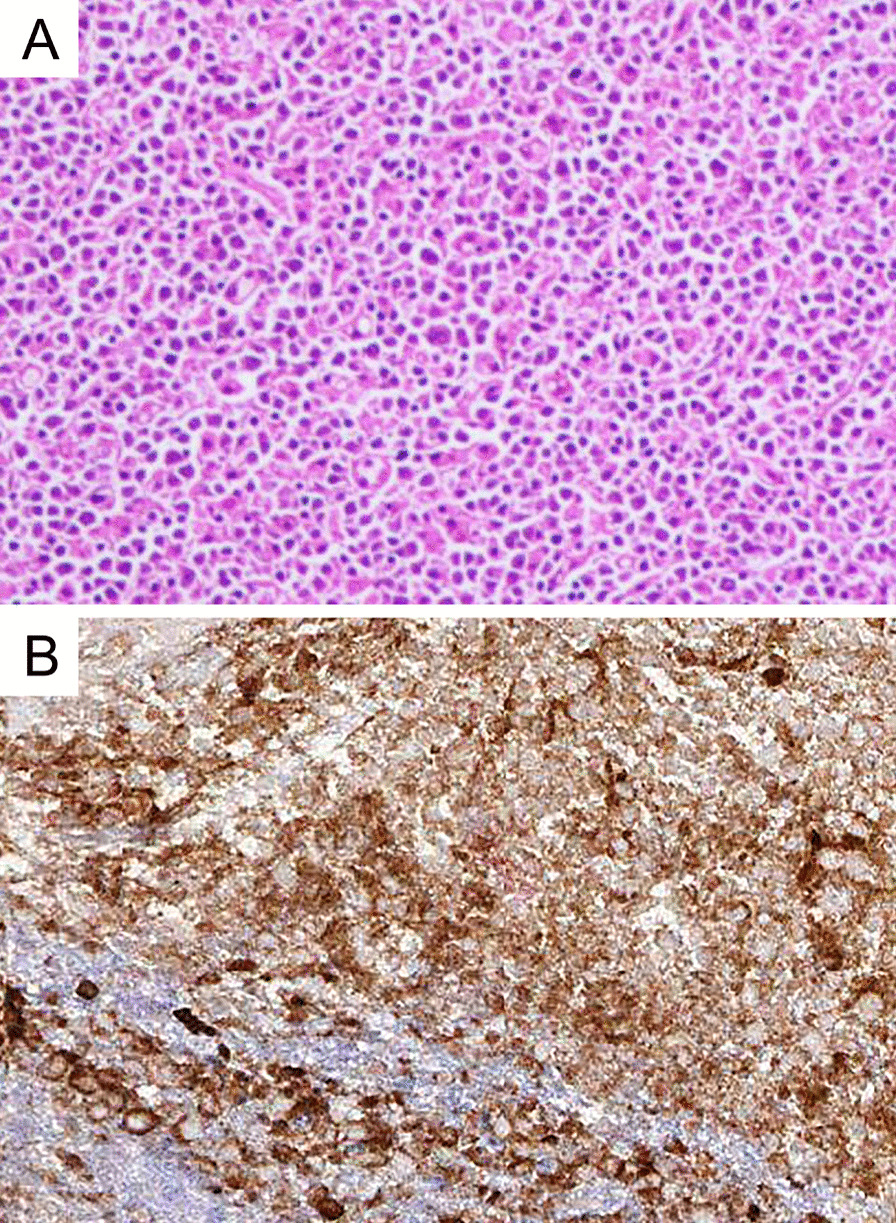
**A** Diffuse growth of large, abnormal lymphocytes in prostate biopsy tissue, suggesting diffuse large B-cell lymphoma [hematoxylin and eosin (HE), ×100]. **B** Immunohistochemistry staining showing positivity for CD20 (×100)

**Fig. 2:**
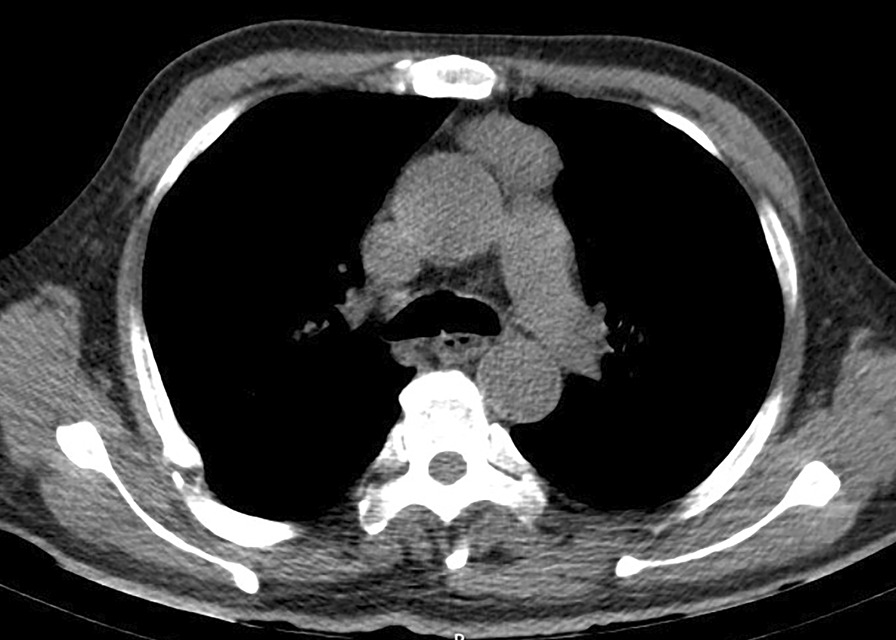
CT scan showing an anterior mediastinum soft tissue shadow

**Fig. 3:**
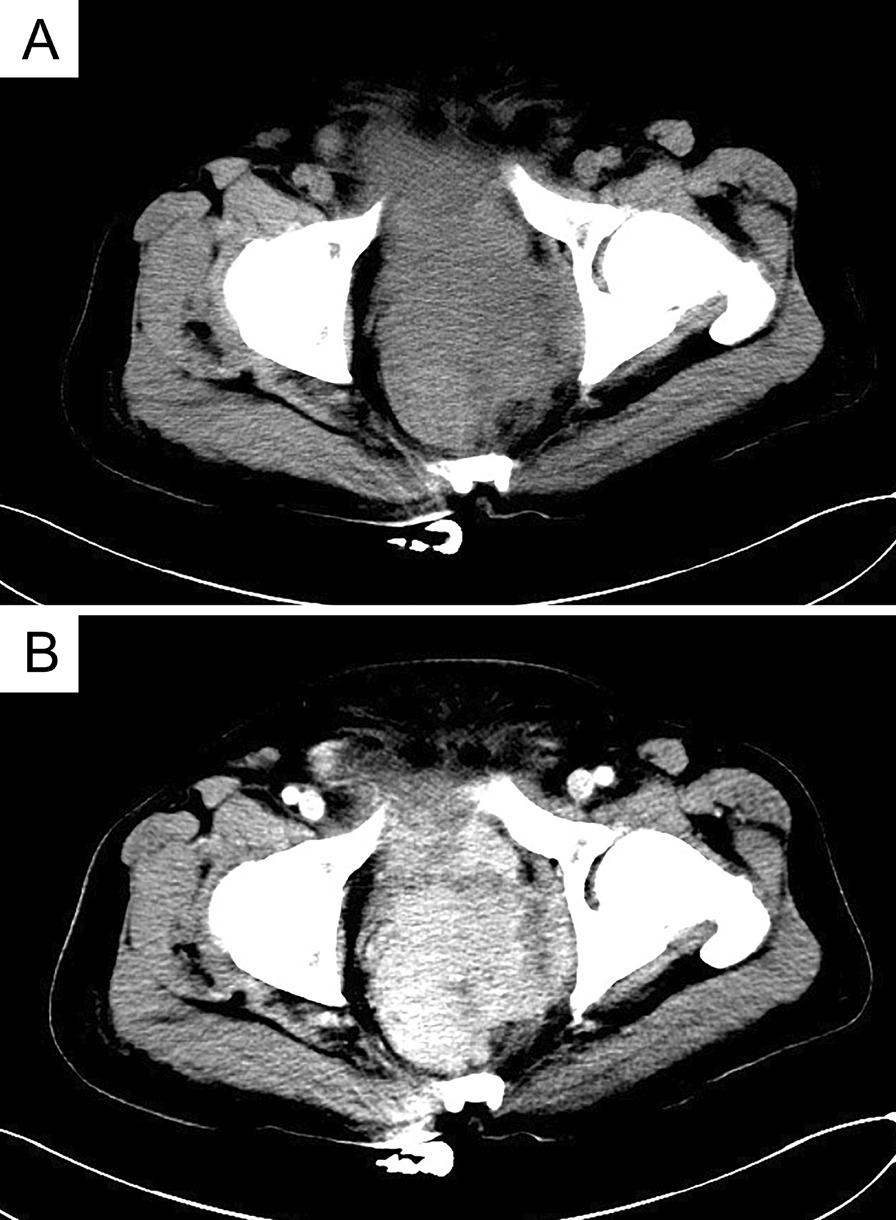
**A** CT scan showing an abnormal morphology of the prostate and bilateral seminal vesicles, with a large mass in the prostate area with unclear boundaries with the posterior wall of the bladder and rectum. **B** Enhancement scan showing significant inhomogeneous enhancement of the mass in the arterial phase

A 70-year-old Asian male patient presented with a 2-year history of intermittent dysuria. He had symptoms of thinner urine flow, shorter range, prolonged urination, and incomplete urination, accompanied by urgency and frequent urination. Initially, because the symptoms were still mild and did not significantly affect daily life, the patient did not pay attention and was not diagnosed and treated systematically. Since May 2019, the above symptoms of the patient were significantly worse than before, and he was admitted to the urology department of our hospital for further treatment. There was no antecedent history of illness or trauma and no family history of malignancies. Physical examination did not reveal any abnormal finding except of diffusely enlarged prostate during rectal examination. Pelvic magnetic resonance imaging (MRI) showed that the peripheral zone of the prostate was occupied, which was considered because prostate cancer (or sarcoma) invades the bilateral seminal vesicle glands, the posterior wall of the bladder, and the anterior wall of the rectum with pelvic lymph node metastasis. However, his serum prostate-specific antigen (PSA) was 0.404 ng/ml, which was within the normal range. A prostate biopsy was performed, identifying a non-Hodgkin lymphoma (Fig. 1) whose immunohistochemical test was positive for antibodies CD20, MUM-1, Bcl-6, CD79a, and CD5 and negative for CD3, CD30, CyclinD1, PSA, P504s, NKX3.1, P63, 34βE12, CgA, Syn, CD56, and CK-pan. Also, Ki-67 staining was 70% positive, showing a high proliferation and invasiveness of the tumor. Gene rearrangement suggested that both immunoglobulin heavy chain (IgH) and T-cell receptor gamma (TCRG) were positive. These testing results supported the diagnosis of diffuse large B-cell lymphoma. At this time, the patient began to have nausea, vomiting, intermittent fever, fatigue, night sweats, difficulty in defecation, and obvious edema of both lower limbs, scrotum, and abdominal wall. The patient was transferred to our department since July 2019 for specialized therapy. Neck, chest, and full-abdomen enhanced computed tomography (CT) revealed an anterior mediastinum soft tissue shadow (Fig. 2), and multiple lymph nodes in the pelvic cavity, retroperitoneal area, and bilateral inguinal areas (Fig. 3), which were all considered to be prostate lymphoma with infiltration of the bladder, seminal vesicles, rectum, and mediastinum. Bone marrow biopsy did not indicate lymphoma infiltration. The patient’s complete blood count results were generally normal, and his lactate dehydrogenase (LDH) test result was 326 U/L, which was above the normal range. The patient received eight courses of chemotherapy with the R-CHOP regimen (including rituximab, cyclophosphamide, doxorubicin, vincristine, and prednisone). Post-chemotherapy CT scans showed complete remission. Following chemotherapy, the patient received intensity-modulated radiotherapy (IMRT) of the prostate with a clinical target volume (CTV) dose of 40 Gy/20 f, including the prostate region. He finished radiation therapy in February 2020 without further treatment, and his condition has stabilized without progression on recent follow-up, with no further symptoms of urinary tract obstruction.

## Discussion

DLBCL is the most common form of non-Hodgkin’s lymphoma, accounting for approximately 30% of all known cases, and up to 40% of DLBCL are developed in the extranodal sites and can be seen anywhere outside the node [2]. Its diagnosis depends on the patient’s clinical symptoms, histopathological manifestations, immunohistochemistry, and relevant molecular test results. Primary lymphoma of the prostate is a relatively rare but well-recognized subtype, while non-Hodgkin lymphoma of the prostate most often presents as a diffuse large B-cell lymphoma [3]. The symptoms appearing are similar to those of other prostate diseases, with the vast majority of patients coming to the hospital for nonspecific symptoms such as urgency, frequency, painful urination, hematuria, increased nocturia, dysuria, and acute urinary retention, while specific symptoms associated with lymphoma such as fever, night sweats, and weight loss rarely appear in the early stages [4].

The earliest diagnostic criteria for primary lymphoma of the prostate, proposed by King and Cox, was that the tumor was confined to the prostate gland and surrounding tissues without any involvement of local lymph nodes [5]. On this basis, Bostwick and Mann defined primary lymphoma of the prostate as: symptoms caused by prostate enlargement; invasion of only the prostate and surrounding connective tissue; after discovery of prostate lesions, no involvement of other sites within 1 month [4]. At present, most clinical work refers to these diagnostic criteria. For the diagnosis of primary lymphoma of the prostate, in addition to routine examinations, urological ultrasound, abdominal and pelvic CT, MRI, positron emission tomography (PET)-CT, whole-body bone scan, cystoscopy, bone marrow biopsy, PSA, LDH, and other examinations are all optional items, which can help to achieve accurate staging and assess the prognosis [6]. Compared with prostate cancer, patients with lymphoma tend to have only a slight or no increase in PSA [4, 7]. The final diagnosis depends on the pathological examination after prostate biopsy.

At present, there is no established consensus on the management for primary lymphoma of the prostate, and there are various treatment methods. Several different treatments have been reported in the established literature, including radical prostatectomy, radiotherapy, chemotherapy, or combinations of chemotherapy and radiotherapy. According to Whitmore, surgical resection can relieve urinary tract obstruction but does not improve survival, rather compromising subsequent chemotherapy, radiation therapy, and quality of life, but when a puncture biopsy is inconclusive and there is a high clinical suspicion of primary lymphoma of the prostate, a surgical biopsy is the best test approach [8]. Radiotherapy is rarely given alone, but it can quickly relieve symptoms of acute urinary tract obstruction and reduce the rate of local recurrence [9, 10]. Chemotherapy is still the primary treatment. Sarris *et al.* suggested the use of an Adriamycin-based chemotherapy regimen based on the histological classification of prostate lymphoma [1]. Most cases reported in recent years were treated with CHOP regimen, and in patients with B-cell lymphoma, the addition of rituximab can improve outcomes [6, 11, 12]. For patients with relapsed or refractory DLBCL, anti-CD19 chimeric antigen receptor T-cell (CAR-T) therapy is an option [13]. Polatuzumab vedotin (Pola), an antibody–drug coupling (ADC) targeting CD79b, has also been shown to be effective in patients with R/R DLBCL who are not candidates for autologous stem cell transplantation (ASCT)[14]. Magrolimab, a macrophage immune checkpoint inhibitor, has shown synergy with rituximab in early studies and is expected to be a new treatment for patients with R/R DLBCL [15]. Other promising agents include Bcl-2 inhibitor venetoclax, BTK inhibitor ibrutinib, and EZH2 inhibitor tazemetostat [16]. However, the efficacy of these new agents alone is limited in patients with DLBCL, and it is unclear whether combination therapy can improve outcomes. Clinical studies have also demonstrated that programmed cell death-1 (PD-1) inhibitors, such as pembrolizumab, are effective in B-cell lymphoma [17], but their value in primary lymphoma of the prostate remains to be further investigated. Following the National Comprehensive Cancer Network (NCCN) guideline of DLBCL, our patient received eight courses of chemotherapy with the R-CHOP regimen and IMRT including the prostate region at a dose of 40 Gy to achieve better clinical outcomes.

There are conflicting reports about the prognosis. Several clinical studies suggested that patients with lymphoma originating in the prostate have a poorer prognosis despite patient age, tumor stage, histologic type, or treatment [8, 18]. In the study of Bostwick and Mann *et al.*, the survival rate of prostate lymphoma was 1 year 64%, 2 years 50%, 5 years 33%, 10 years 33%, and 15 years 16% [4]. Tong Fang summarized 29 cases of primary lymphoma of the prostate; 9 of them died, with a median overall survival of 23 months, while 7 of them had been alive for more than 5 years at the end of the study [7]. Most clinical studies still evaluate the prognosis of prostate lymphoma patients according to the international prognostic indicator (IPI), including five independent prognostic factors: age > 60 years, serum LDH higher than normal, performance status 2–4, stage III or IV, and extranodal involvement at more than one site [19]. The more risk factors, the lower the survival rate. Whether there are adverse prognostic factors applicable to primary lymphoma of the prostate alone requires more case studies.

## Conclusion

Primary diffuse large B-cell lymphoma of the prostate is a rare and highly aggressive disease. Due to its nonspecific symptoms, it is easy to be misdiagnosed as other prostate conditions before a surgical biopsy. This also reminds us that the possibility of this disease should always be considered in the differential diagnosis. Although the prognosis of primary lymphoma of the prostate is generally considered to be poor, the disease can be controlled effectively in most patients with the application of chemotherapy and local radiotherapy. As clinical research continues, we will be able to provide a more rational individualized treatment plan for these patients and achieve better therapeutic outcomes.

## Data Availability

The published information is available from the corresponding author on reasonable request.
